# Supplementary material for: Modelling Skylarks (Alauda arvensis) to Predict Impacts of Changes in Land Management and Policy: Development and Testing of an Agent-Based Model
Source: PLoS One. 2013 Jun 6;8(6):e65803. doi: 10.1371/journal.pone.0065803 (PMC3675089; doi:10.1371/journal.pone.0065803)
Supplement: Supporting Information S4 — The skylark ODdox as a zipped archive. (ZIP) [file pone.0065803.s004.zip › Skylark_ODdox/functions.html]

ALMaSS Skylark ODdox: Class Members


ALMaSS Skylark ODdox
 2.0


- Main Page
- Related Pages
- Classes
- Files

- Class List
- Class Index
- Class Hierarchy
- Class Members

- All
- Functions
- Variables
- Typedefs

- a
- b
- c
- d
- e
- f
- g
- h
- i
- j
- k
- l
- m
- n
- o
- p
- r
- s
- t
- u
- v
- w
- x
- y
- z
- ~

Here is a list of all class members with links to the classes they belong to:

### - a -

- ActivePit()
  : ActivePit
- AddArea()
  : LE
- AddBeetleBanks()
  : Landscape
- AddEgg()
  : Skylark\_Clutch
- AddField()
  : FarmManager
  , Farm
- AddGreenElement()
  : Landscape
- AddNewEvent()
  : Farm
- AddPolygon()
  : PolygonDataVector
- AddStriglingMort()
  : Skylark\_Population\_Manager
  , Skylark\_Base
- AddToClusterList()
  : Landscape
- Age
  : Skylark\_Base
- age
  : PreFledgeling\_struct
  , AdultSkylark\_struct
- AgroChemIndustryCerealFarm1()
  : AgroChemIndustryCerealFarm1
- AgroChemIndustryCerealFarm2()
  : AgroChemIndustryCerealFarm2
- AgroChemIndustryCerealFarm3()
  : AgroChemIndustryCerealFarm3
- AlFreq
  : Population\_Manager
- AmenityGrass()
  : AmenityGrass
- AssignPermanentCrop()
  : UserDefinedFarm
- AutumnHarrow()
  : Farm
- AutumnPlough()
  : Farm
- AutumnRoll()
  : Farm
- AutumnSow()
  : Farm
- AxisLoop()
  : Landscape


- Generated on Thu Jan 10 2013 13:15:39 for ALMaSS Skylark ODdox by
   1.8.1.1
